# Natural Products in Anti-Obesity Therapy

**DOI:** 10.3390/molecules21121750

**Published:** 2016-12-20

**Authors:** Filomena Conforti, Min-Hsiung Pan

**Affiliations:** 1 Department of Pharmacy, Health and Nutritional Sciences, University of Calabria, via Pietro Bucci, 87036 Arcavacata di Rende (CS), Italy; filomena.conforti@unical.it; 2 Institute of Food Science and Technology, National Taiwan University, Taipei 10617, Taiwan

Obesity is regulated by genetic, endocrine, metabolic, neurological, pharmacological, environmental, and nutritional factors. The brain, gut, and adipose tissues interact with each other through metabolism-related neuropeptides, cytokines, chemokines, adipokines, and gut microbial composition. This contributes to changes in energy intake and energy expenditure. Globally, the increasing incidence of obesity is disturbing. In recent decades, obesity has been recognized as a chronic disease and a serious public health issue. It is well established that obesity causes a lifetime risk of various metabolic disorders such as dyslipidemia, hypertension, type II diabetes mellitus, cardiovascular diseases, and certain types of cancer. These are also associated with hyperglycemia, high cholesterol, insulin resistance, and fatty liver disease. Therefore, early prevention of the occurrence of obesity is necessary.

In order to fight obesity, a balanced diet, healthy lifestyle, and pharmacological therapies plus moderate-intensity exercise have been highly praised. Notably, the intake of functional foods is the most ideal treatment modality for weight loss as natural products are non-toxic and healthy. This Special Issue is a collection of seven reviews and seven research articles on the preclinical and clinical benefits of natural products in controlling obesity. The research articles in this issue are broadly divided into four product sources—herbs, fruits, beverages, and *trans*-fatty acids.

Six research articles of this Special Issue focus on evaluating the preventive role and regulated mechanisms of natural products on obesity, insulin resistance, and hepatic steatosis (fatty liver disease). Ansari et al. [1] demonstrated that the detailed molecular mechanisms of *Chowiseungcheng-tang* (CST), an herbal formulation, are beneficial for anti-obesity by modulating metabolism-related neuropeptides, adipokines, and gut microbial composition. The benefits of other traditional herbal medicines including herbal formula HT048 and Zicao (*Lithospermum erythrorhizon*) on high-fat diet (HFD)-induced obese rats and spontaneously obese *db/db* mice was also reported. Lee et al. [2] suggested that HT048 taken as dietary supplement helps to decrease obesity and insulin resistance. Su et al. [3] provided the evidence that the main ingredient of Zicao, Acetylshikonin (AS), exerts anti-obesity and anti-nonalcoholic fatty liver disease (NAFLD) efficacies through the regulation of lipid metabolism and anti-inflammatory effects.

In an interesting article, Tung et al. [4] elucidated that piceatannol (3,3’,4,5’-tetrahydroxy-*trans*-stilbene; Pic), an analogue and metabolite of resveratrol (Res) obtained from red wine, grapes, blueberries, and passion fruits, is shown to decrease lipid accumulation in adipocytes and the liver via the regulation of AMPK expression and gut microbiota. Further, *Poncirus trifoliata* L. fruit extracts (flavedo (PF) and juice sacs (PJ)) were also found to influence lipid and glucose metabolism and exhibited anti-obesity and hypoglycemic effects [5]. Pu-erh tea, a traditional Chinese beverage, has been believed to have numerous health benefits. This is the reason why Xiao et al. [6] took the advantages of RNA-Seq to determine the gene expression profiling and transcriptional characters of the Pu-erh tea treated *C. elegans* and found that the *vit* family is responsive for Pu-erh tea’s function of reducing fat accumulation.

However, one of the research articles by Zhao et al. [7] confirms that consumption of a diet high in *trans*-fatty acids (uncommon in nature) induces higher rates of obesity, insulin resistance (IR), and hepatic steatosis in male C57BL/6 mice, possibly by suppressing the IRS1, diacylglycerol acyl synthetase (DGAT1), adipose triglyceride lipase (ATGL), and acyl-CoA synthetase (ACSL1) dependent pathways.

This Special Issue also contains seven review articles that focus on the anti-obesity potential of various tea polyphenols, plant extracts, herbal products, phytochemicals, and tocotrienols on IR which leads to obesity and metabolic complications. The reviews by Pan et al. [8] and Suzuki et al. [9] provide an overview on the recent data highlighting the favorable effects of black tea, green tea, and its catechins in lipid and saccharide digestion, absorption and intake, promotion of lipid metabolism, blockage of the pathological processes of obesity, and comorbidities of obesity by reducing oxidative stress and effects on intestinal microbiota. Two reviews by Avalos-Soriano et al. [10] and Marrelli et al. [11] investigated a detailed account on molecular regulation of the insulinotropic and insulin-sensitizing activity and anti-obesity therapeutic potential by 4-Hydroxyisoleucine (4-OHIle) and saponins isolated from fenugreek (*Trigonella foenum-graecum*) seeds and medicinal plants. In this Special Issue, two other reviews also summarized the anti-obesity effects of different dietary and herbal natural products, their active ingredients, and anti-adipogenesis mechanisms of action [12,13]. To recognize the physical properties of tocotrienols (T3s) and assess their isomers among T3s potential in regulating obesity, Zhao et al. [14] comprehensively reviewed the scientific literature regarding the impact of T3s on obesity with a particular emphasis on the signaling pathways involved.

Overall, we hope this Special Issue will enhance your knowledge of the anti-obesity effects of natural products, provide effective therapeutic strategies, and attract the reader’s interest in developing novel and safe anti-obesity drugs.
